# Improved Mitochondrial Function Underlies the Protective Effect of Pirfenidone against Tubulointerstitial Fibrosis in 5/6 Nephrectomized Rats

**DOI:** 10.1371/journal.pone.0083593

**Published:** 2013-12-09

**Authors:** Jun-Feng Chen, Hong Liu, Hai-Feng Ni, Lin-Li Lv, Ming-Hui Zhang, Ai-Hua Zhang, Ri-Ning Tang, Ping-Sheng Chen, Bi-Cheng Liu

**Affiliations:** 1 Institute of Nephrology, Zhong da Hospital, Southeast University School of Medicine, Nanjing, Jiangsu, China; 2 Department of Nephrology, Nanjing Children’s Hospital, Nanjing Medical University, Nanjing, Jiangsu, China; 3 Institute of Pediatrics, Nanjing Medical University, Nanjing, Jiangsu, China; 4 Department of Pathology, Southeast University School of Medicine, Nanjing, Jiangsu, China; Baker IDI Heart and Diabetes Institute, Australia

## Abstract

Dysfunctional mitochondria participate in the progression of chronic kidney disease (CKD). Pirfenidone is a newly identified anti-fibrotic drug. However, its mechanism remains unclear. Mitochondrial dysfunction is an early event that occurs prior to the onset of renal fibrosis. In this context, we investigated the protective effect of pirfenidone on mitochondria and its relevance to apoptosis and oxidative stress in renal proximal tubular cells. A remnant kidney rat model was established. Human renal proximal tubular epithelial cells (HK2) using rotenone, a mitochondrial respiratory chain complex Ι inhibitor were further investigated in vitro to examine the mitochondrial protective effect of pirfenidone. Pirfenidone protected mitochondrial structures and functions by stabilizing the mitochondrial membrane potential, maintaining ATP production and improving the mitochondrial DNA (mtDNA) copy number. Pirfenidone decreased tubular cell apoptosis by inhibiting the mitochondrial apoptotic signaling pathway. Pirfenidone also reduced oxidative stress by enhancing manganese superoxide dismutase (Mn-SOD) and inhibiting intracellular reactive oxygen species (ROS) generation, which suggested that the anti-oxidant effects occurred at least partially via the mitochondrial pathway. Pirfenidone may be effective prior to the onset of renal fibrosis because this drug exerts its anti-fibrotic effect by protection of mitochondria in renal proximal tubular cells.

## Introduction

 Chronic kidney disease (CKD) is a major public health problem that imposes enormous socioeconomic burdens on patients, families and societies. Renal fibrosis, particularly tubulointerstitial fibrosis, is a common final outcome of most progressive CKD [1]. Major cellular events in tubulointerstitial fibrosis include inflammatory cell infiltration, fibroblast activation, the loss of peritubular capillaries and tubular atrophy [2]. Mitochondria are energy-producing organelles that perform key cellular tasks. A deregulation of the mitochondrial respiratory machinery was observed in patients with CKD [3]. The structural characteristics of CKD may be partially due to the gradual loss of renal energy through the development of mitochondrial dysfunction. Dysfunctional mitochondria contribute to the pathophysiology of renal disease [4]. Yuan [5] et al demonstrated that mitochondrial dysfunction of renal proximal tubular epithelial cells is involved in the pathogenesis of epithelial-mesenchymal transition (EMT). Manoli [6] et al also suggested that proximal tubular mitochondrial dysfunction was a key pathogenic mechanism of methylmalonic academia (MMA)-associated kidney disease. Therefore, the restoration of mitochondrial function is clearly beneficial for the treatment of CKD. 

The increase in renal tubular epithelial cell apoptosis is an important characteristic of tubulointerstitial fibrosis. Renal tubular epithelial cell apoptosis is a critical detrimental event that leads to chronic kidney injury, which is associated with renal fibrosis [7]. Mitochondria are the core of the signaling cascade of the intrinsic apoptosis pathway [8], and mitochondrial dysfunction increases tubular cell apoptosis [9]. Oxidative stress develops from an imbalance in free radical production, which increases through dysfunctional mitochondria [3], and oxidative stress in the kidney contributes to renal fibrosis [10]. Therefore, an effective therapy to protect mitochondrial function in renal tubular cells after injury may counteract apoptosis and oxidative stress, which are implicated in the inhibition of renal fibrosis. 

 Pirfenidone (5-methyl-1-phenyl-2(1H)-pyridone), is an oral derivative of pyridine that exhibits anti-fibrotic properties in fibrotic diseases. However, the precise mechanism of pirfenidone is not completely understood. Shihab [11] et al demonstrated that pirfenidone significantly decreased apoptosis-positive cells and down-regulated pro-apoptotic gene expression in a chronic cyclosporine A (CsA) nephrotoxicity rat model, which suggested that the anti-fibrotic properties of pirfenidone were partially exerted through anti-apoptotic mechanisms. However, the protective effect of pirfenidone on mitochondrial function in the kidney is not clear.

This study investigated the protective properties of pirfenidone in renal tubular epithelial cell mitochondria in a 5/6 nephrectomized rat model. The anti-apoptotic and anti-oxidative effects of pirfenidone were also examined in vivo and in vitro. Our study provided a novel mechanism of pirfenidone action for the treatment of renal fibrosis.

## Materials and Methods

### Animals

Six-week-old male Sprague-Dawley (SD) rats weighing 180-200g were purchased from Shanghai Slac Laboratory Animal Co., Ltd. (Shanghai, China). The rats were maintained under stable room temperature and a regular 12 h dark and light rhythm for one week prior to the experiments. The rats were fed standard rat feed and had free access to tap water. All animals received humane care in compliance with university guidelines. The Ethics Review Committees for Animal Experimentation of Southeast University approved the experimental protocol (Permit Number: 0098).

### Surgical Procedure and Drug Administration

Each rat was anesthetized with a chloral hydrate solution (33mg/100g) via intraperitoneal injection. Twenty rats underwent a five-sixths nephrectomy in which the right kidney was removed and two-thirds of the left kidney was ablated. A sham operation was performed on ten additional rats as a non-nephrectomized control (sham).

 Pirfenidone (Licheng Chemical Co., Ltd, Shanghai, China) was suspended in a 0.5% carboxymethylcellulose solution (vehicle). Nephrectomized rats were randomly divided into two groups: without treatment (control, n=10) and treatment with pirfenidone (n=10). Pirfenidone (500mg/kg/d) was administered daily by gavage for twelve weeks until sacrifice, whereas the vehicle was administered to the nephrectomized rats without treatment and the sham group.

### Biochemical Analysis

Twenty-four hour urinary protein and N-acetyl-β-D-glycosaminidase (NAG) activity were determined at the 12^th^ week post-surgery. All rats were sacrificed 12 weeks after surgery, and sera were collected. A biuret assay was used to determine the 24-h urinary protein, the procedures were as follows: add Sulfuric acid and Sodium tungstate solution to urine samples. After 10 min, centrifuge at 300 rpm for 10 min. discard the supernatant fluid, dissolve the precipitation with distilled water. Add biuret reagent to each tube, vortex immediately, and incubated at 37°C for 30 min. Read at 540nm. NAG activity was determined using an assay kit (Jiancheng Bioengineering Institute, Nanjing, China). In brief, this method uses the substrate 3-cresolsulfonphthaleinyl-N-acetyl-β-D-glucosaminidine-sodium, which is hydrolyzed by NAG when present in the urine. 0.5ml of the substrate solution was incubated at 37°C for 5 min. A 100μl aliquot of the urinary sample was mixed and incubated at 37°C for 15 min. After incubation, 2ml of the stop reagent solution was added to the sample mixture. The absorbance was measured by a spectrophotometer (Shimazu, Tokyo, Japan) set at 400 nm. Serum urea nitrogen (BUN) and creatinine (Scr) were determined using automatic analyzers (Hitachi, Tokyo, Japan). 

### Kidney Histopathology

The partial kidneys were collected and weighed. The renal tissues were fixed in 10% buffered formalin and embedded in paraffin. Sections (2-4μm thick) were stained with periodic acid-schiff (PAS) reagent and trichrome masson stain and observed under light microscopy. Renal interstitial fibrosis was scored in 20 randomly selected non-overlapping specimens from each rat at 200×magnification on trichrome-masson staining according to the protocol described previously [12,13].

### TdT-mediated dUTP nick end labeling (TUNEL) Assay for Apoptosis

The tissue sections were prepared according to standard protocols. The slides were incubated with proteinase K for 30 min at room temperature. TUNEL labeling was performed using an *in situ* cell death detection kit (Roche, Basel, Switzerland) according to the manufacturer’s instructions. The apoptotic cells in the proximal tubules were quantified in thirty random fields in each sample, and an apoptosis index (AI) was calculated and averaged. 

### Cell Culture

Human renal proximal tubular epithelial cells (HK2) were purchased from China Center for Type Culture Collection (WuHan, China). Cells were cultured in DMEM/ F12 media (Gibco, UK) supplemented with 10% heat-inactivated fetal calf serum (Gibco, UK). The medium was replaced when confluent with serum-free medium for 24 h prior to the start of the experiment. 

 Rotenone as a mitochondrial respiratory chain complexes Ι inhibitor was used. This inhibitor was purchased from the Sigma Company and dissolved in DMSO. HK2 cells were incubated with rotenone (2μM) for 48 h to induce cell apoptosis with or without pirfenidone (100, 500 and 1000μg/ml) intervention. 

### Cell Viability

 Cells (in 96-well plates) were analyzed for cell viability using a Cell Counting Kit-8 (CCK-8) assay (Dojindo, Japan). The cells were resuspended and incubated for the indicated times at 37°C in a 5% CO_2_ incubator. A CCK-8 solution (10μl) was added to the cell suspension and incubated for 1 h. The absorbance at 450 nm was measured using a microplate reader. Cell viability was calculated and averaged. 

### Cell Death Detection Assay

The Annexin V-FITC apoptosis detection kit (Invitrogen, USA) detected apoptotic cells according to manufacturer’s protocol. Briefly, the cells were washed with PBS and collected using trypsinization. The cells (1×10^6^ cells) were resuspended in 1×binding buffer, incubated with 5μl of Annexin V-FITC and 10μl of the PI solution and incubated for 15 min in the dark. The apoptotic cells were determined immediately using flow cytometry (BD, USA), which measured the fluorescence at 488 nm excitation and 530 nm emission. The apoptotic cells were quantified, and the percentage of apoptotic cells was calculated. 

### Isolation of Mitochondria and Cytosolic Fractions

Remnant kidney cortexes were cut into pieces and incubated in isolation buffer (75 mM sucrose, 225 mM mannitol, 5 mM MgCl_2_, 5 mM KH_2_PO_4_, 1 mM EGTA, 0.1 mM PMSF, 5 mM HEPES-KOH, pH 7.4), homogenized for 3 min, and centrifuged at 600 g for 5 min at 4°C. The supernatants were centrifuged at 11,000 g for 10 min at 4°C to obtain the mitochondrial pellets. Cytosolic fractions were obtained after further centrifugation at 12,000 g for 10 min at 4°C. The protein concentrations of the supernatants and mitochondrial fractions were measured for western blotting analysis.

HK2 cells were washed twice in PBS and resuspended in extract buffer (20 mM Hepes-KOH, 1.5 mM MgCl_2_, 1 mM EDTA, 1 mM EGTA, 1 mM DTT, and 0.1 mM PMSF, pH 7.5). The resuspended cells were homogenized, and the homogenates were centrifuged twice at 600 g for 10 min at 4°C. The supernatants were centrifuged at 11,000 g for 10 min at 4°C to obtain the mitochondrial pellets. Cytosolic fractions were obtained after further centrifugation at 12,000 g for 10 min at 4°C. The protein concentrations of the supernatants and mitochondrial fractions were measured for further analysis. 

### Electron Microscopy

 The pieces of the renal cortex and the HK2 cells were fixed in 4% paraformaldehyde, postfixed in 1% osmium tetroxide, dehydrated in graded alcohols, and embedded in epoxy resin. Ultrathin sections were stained with uranyl acetate/lead citrate and examined using transmission electron microscopy (JEOL, Tokyo, Japan). Flameng score [14] was used to evaluate degree of injury of mitochondria. In each slice, five visual fields were selected randomly. In each field, 20 mitochondria were chosen at random. 

### Mitochondrial Membrane Potential (MMP) Measurement

The mitochondrial membrane potential (∆Ψm) was measured using 5,-5',-6, 6'-tetrachloro-1,-1-',-3,-3-'-tetrethyl benzimidalyl carbocyanine iodide (JC-1, Invitrogen). Renal isolated mitochondria or HK2 cells were incubated with 10μg/ml of JC-1 at 37°C for 20 min and washed three times in PBS. The fluorescence intensity was measured using red (excitation 525 nm/emission 590 nm) and green (excitation 488 nm/emission 525 nm) wavelengths using a fluorescence multimode microplate reader (Tecan Infinite 200 PRO, Switzerland) for renal isolated mitochondria and flow cytometry (BD, USA) for HK2 cells. The results were calculated as the ratio of red to green fluorescence. Carbonyl cyanide m-chlorophenylhydrazone (CCCP) (10μM) was used in HK2 cells as a positive control. 

### Mitochondrial and Intracellular reactive oxygen species (ROS) Measurement

ROS was measured using 2',-7'-dichlorodihydrofluorescein diacetate (H_2_DCFDA, Invitrogen). Wash the renal isolated mitocdhondria or cultured HK2 cells 3 times with PBS. Then, Renal isolated mitochondria or HK2 cells were incubated with 10μM H_2_DCFDA at 37°C for 30 min. Excess H_2_DCFDA was removed by PBS for 4 times, and the mitochondrial or cellular fluorescence of dichlorofluorescein (DCF) was determined at 485 nm excitation and 538 nm emission in a fluorescence multimode microplate reader (Tecan Infinite 200 PRO, Switzerland). Rosup (50mg/ml) was used as a positive control. Hydrogen peroxide released by HK2 cells was assessed in a reaction mixture containing Amplex Red (Invitrogen, USA) and was determined at 560 nm in a microplate reader according to the manufacturer’s instruction. In brief, prepare a reaction mixture containing 50μM Amplex Red reagent and 0.1U/ml HRP in Krebs-Ringer phosphate (KRPG). Pipet 100μL of the reaction mixture into each microplate well and prewarm the reaction mixture at 37°C for 10 min. Add 1.5×10^4^ cells in 20μL of KRPG. A fluorescence microplate reader (Tecan Infinite 200 PRO, Switzerland) was used at excitation 530 nm and emission 590 nm. H_2_O_2_ working solution (20mM ) diluted to 10μM in 1X reaction buffer was used as a positive control. The relative units of DCF fluorescence determined by Amplex Red from different samples are reported as the means±SEM .A standard curve was used to determine H_2_O_2_ concentrations. Dilute the appropriate amount of 20mM H_2_O_2_ working solution into 1X Reaction Buffer to produce H_2_O_2_ concentrations of 0, 2, 4, 6, 8 and 10 μM, each in a volume of 50 μL. 

### ATP Content Assay

 The ATP Colorimetric/Fluorometric Assay Kit (Biovision, USA) was used to detect the ATP content in the kidney cortex and HK2 cells according to the manufacturer’s protocol. Renal tissues or HK2 cells were collected after washing in PBS and lysed in 100μl of ATP assay buffer. The lysates were centrifuged at 15,000 g for 2 min at 4°C. The supernatants were collected and added to a final volume of 50μl per well in a 96-well plate followed by incubation at RT for 30 min. OD values were measured at 570 nm using a fluorescence multimode microplate reader (Tecan Infinite 200 PRO, Switzerland). The ATP content was calculated from the standard curve. 

### Quantitative real-time PCR

Total DNA from kidney cortexes and HK2 cells were extracted using the DNeasy tissue kit (Qiagen, Valencia, USA). Real-time PCR detected mitochondrial DNA (mtDNA) copy number. Reactions were performed in an ABI PRISM 7300 real-time PCR system (Applied Biosystems, CA). The primer sequences were used as followings: Rats: mtDNA , Forward (5′-3′) TCCTCCGTGAAATCAACAACC /Reverse (5′-3′) GGGAACGTATGGACGATGAAC; 18s rRNA, Forward (5′-3′) GCGG TTCT ATTTT GTTG G TTTT/Reverse(5′-3′) A CCTCCGACTTTCGTT CTTG. HK2 Cells: mtDNA, Forward (5′-3′) CGATTCTTTACCTTTCACTTCAT CTT/Reverse(5′-3′) GAGGGCGTCTTTGATTGTGT; 18s rRNA Forward (5′-3′) GCGGTTCTATTTTGTTGGTTTT/Reverse (5′-3′) ACCT CCGA CTTTCGTTCTT G.18S rRNA served as controls for mtDNA for reaction efficiency. The results were analyzed using the comparative cycle threshold (∆∆Ct) method. 

### Western blotting

The mitochondrial and cytosolic fractions of the kidney cortexes and HK2 cells were separated using sodium dodecyl sulfate polyacrylamide gel electrophoresis, and transferred to polyvinylidene difluoride (PVDF) membranes (Millipore, MA, USA). Skim milk (5%) was applied to block nonspecific antibody binding for 1 h at room temperature. The membranes were incubated with primary antibodies against prohibitin (Santa Cruz, USA), cytochrome C (Santa Cruz, USA), cleaved caspase-3 (Abcam, UK) and cleaved caspase-9 (Abcam, UK) overnight at 4°C, followed by incubation with peroxidase-conjugated secondary antibody. The proteins were detected using an ECL advanced system (GE Healthcare, Chalfont St. Giles, UK). 

### SOD, MDA and GSH assay

Renal cortical tissues were homogenized, and HK2 cells lysates were centrifuged for supernatant collection. The enzyme activities of total superoxide dismutase (TSOD), malonaldehyde (MDA) and glutathione (GSH) were determined in the supernatants. SOD, MDA and GSH assays were performed according to the manufacturer’s instructions (Jiancheng Bioengineering Institute, Nanjing, China). SOD activity was determined as the inhibition of the reduction of nitrol blue tetrazolium in the sample at 550 nm. Mn-SOD activity was calculated as the measurement of total SOD and Cu, Zn-SOD activity. MDA levels were analyzed with 2-thiobarbituric acid. GSH levels were measured using the DTNB-GSSG reductase recycling assay. All of the assays used colorimetric methods based on biochemical reactions, and the results were calculated using the absorbance values of the samples and standard equations. 

### Correlation Analysis

 Correlations between renal interstitial fibrosis and renal tubular epithelial cells apoptosis in nephrectomized rats with and without pirfenidone treatment were analyzed using the Spearman rank-order correlation. *P*<0.05 was considered statistically significant. 

### Statistical analysis

 All results are expressed as the means±SEM. Comparisons between groups were performed using one-way analysis of variance (ANOVA) in SPSS 17.0. *P* values <0.05 were considered significant. 

## Results

### Pirfenidone improved biochemical parameters in rats

**Figure 1:**
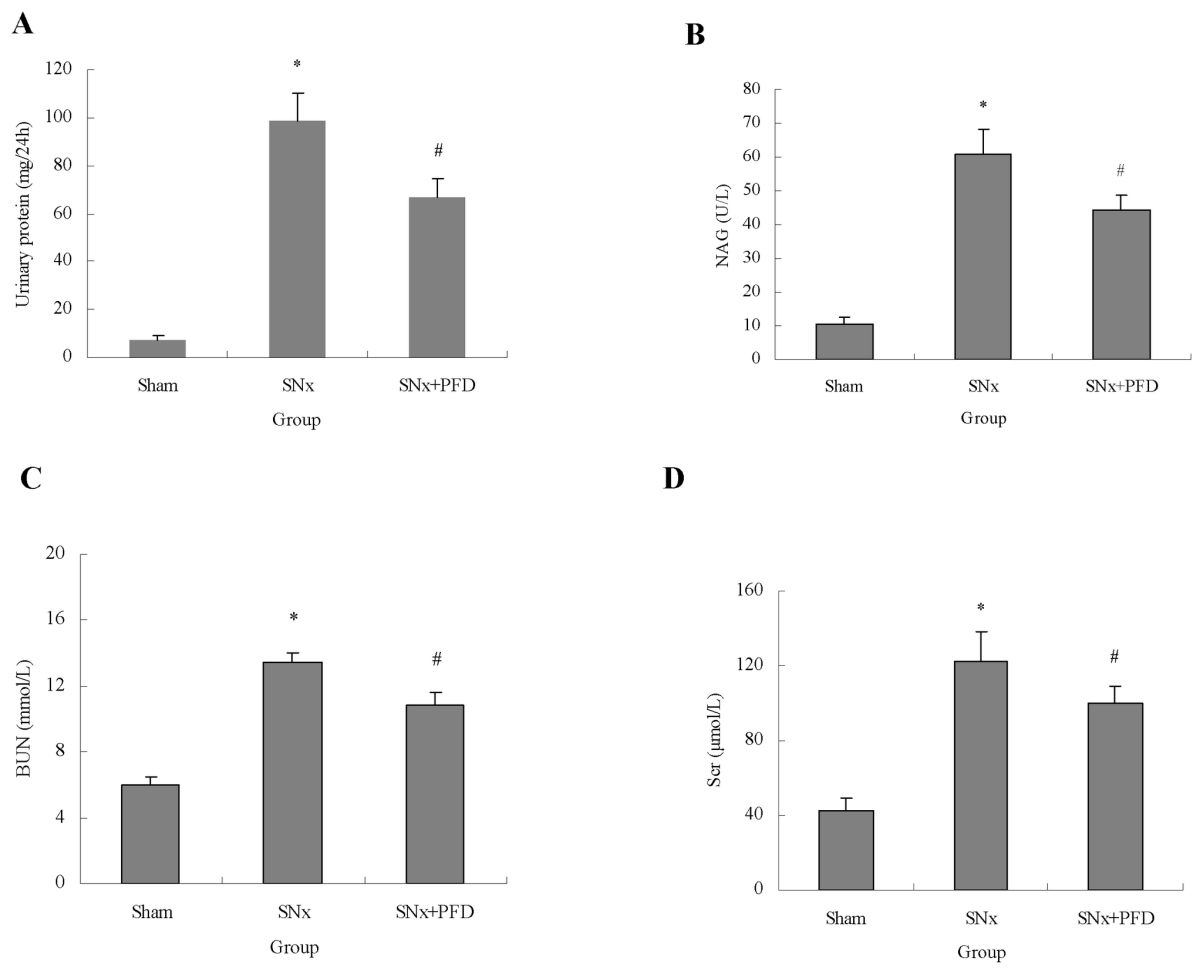
Pirfenidone improved general parameters in SNx rats. Urinary protein excretion (A), NAG activity (B), blood urea nitrogen (C) and serum creatinine in sham-operated, SNx and PFD-treated rats at the 12^th^ week post-surgery. NAG, N-acetyl-β-D-glycosaminidase; BUN, blood urea nitrogen; Scr, serum creatinine; SNx, 5/6 nephrectomy; PFD, pirfenidone. Values are represented as the means±SEM (n=10). ^*^ *P*<0.05, SNx vs. sham; ^#^ *P*<0.05, SNx+PFD vs. SNx rats.

A remnant rat model was established, and rats were treated with pirfenidone for 12 weeks. Pirfenidone significantly decreased the levels of 24-h proteinuria and NAG activity (Figure 1 A and B). Renal functions, including BUN and Scr, were also improved after pirfenidone treatment (Figure 1 C and D). 

### Pirfenidone ameliorated mitochondrial injury in proximal tubular cells in rats

**Figure 2:**
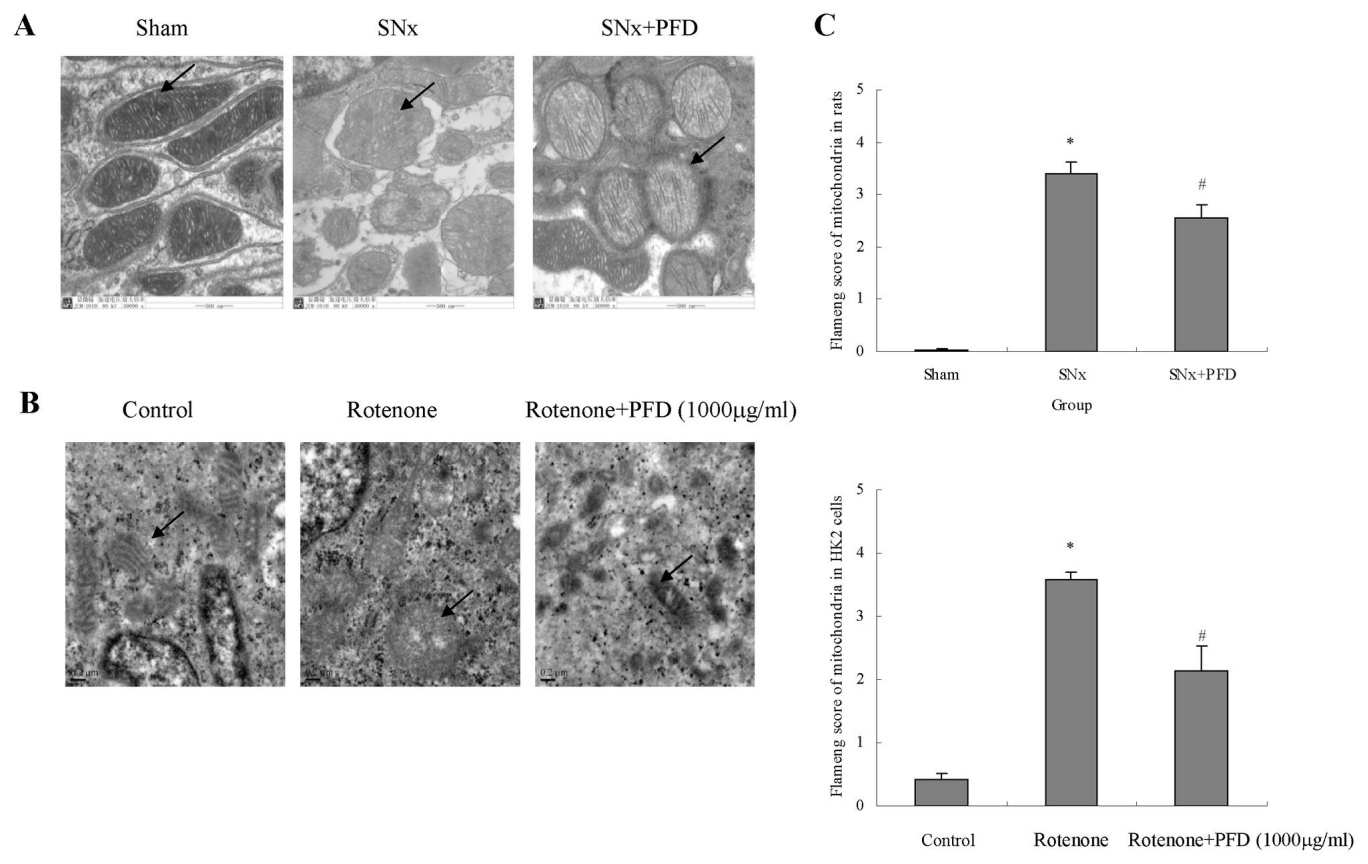
Pirfenidone protected renal proximal tubular cell mitochondrial structure. (A) Representative images of mitochondria in renal proximal tubular cells in rats using transmission electron microscopy. Bars=500nm. (B) Representative images of HK2 cells using transmission electron microscopy. Arrows indicate the mitochondria in different groups. Bars=0.2μm. (C) Flameng grading of injury of renal proximal tubular mitochondria in SNx rats or HK2 cells. SNx, 5/6 nephrectomy; PFD, pirfenidone.

**Figure 3:**
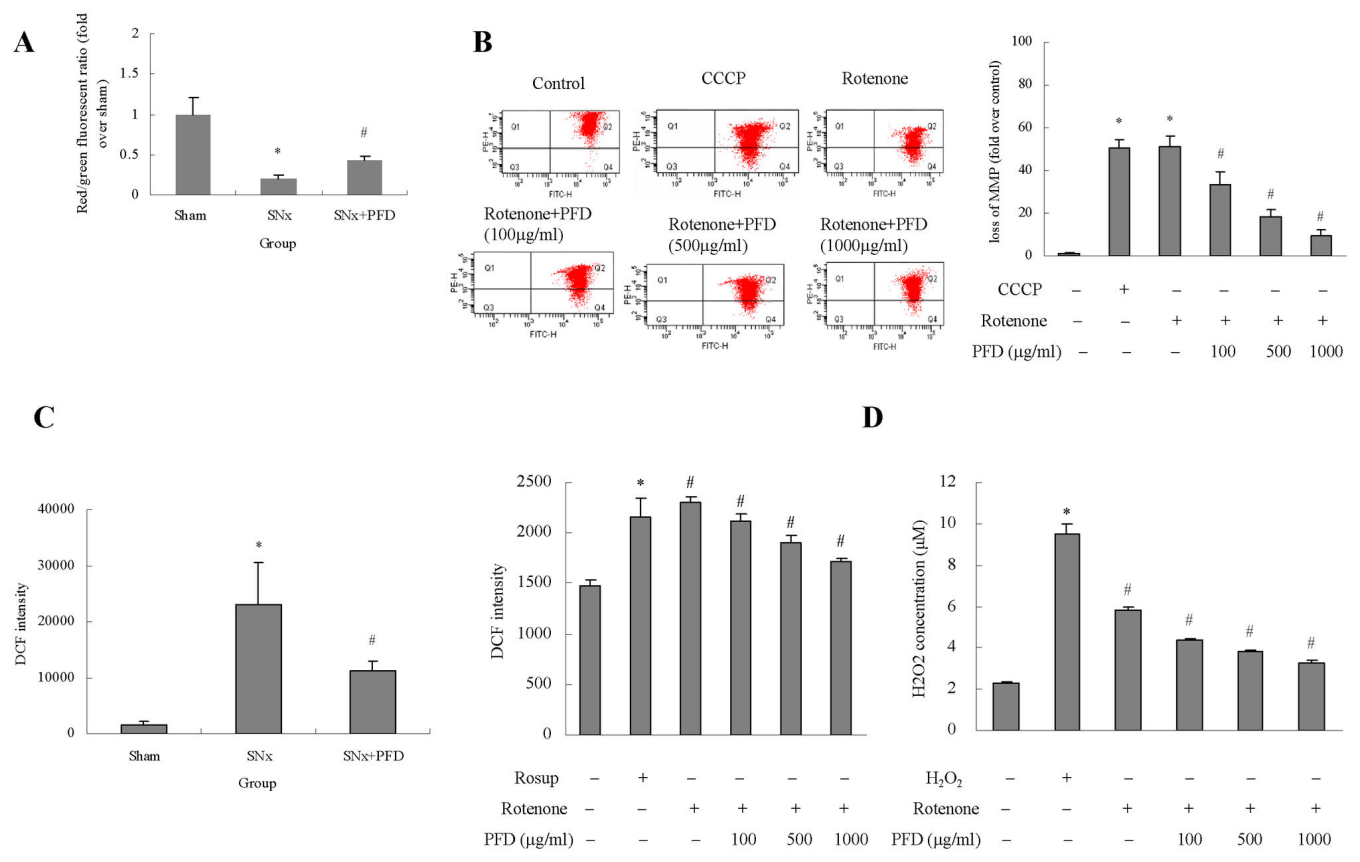
Pirfenidone stabilized the mitochondrial membrane potential (MMP) and decreased intracellular ROS in renal proximal tubular cells. (A) Quantitative analysis of MMP in the kidney cortex of rats. Values are represented as the means±SEM (n=10). (B) MMP in HK2 cells was measured using flow cytometry after JC-1 staining; Quantitative analysis of MMP deterioration in HK2 cells. Values are represented as the means±SEM of three replicates from three different experiments. CCCP was used as a positive control. (C) Dichlorofluorescein (DCF) fluorescence was determined using a fluorescence multimode microplate reader. Quantitative analysis of DCF in the kidney cortex of rats or HK2 cells. Rosup (50mg/ml) was used as a positive control in HK2 cells. (D) H_2_O_2_ release by HK2 cells was measured in 50μl cell culture media preincubated with Amplex Red reagent for 30 min at room temperature. Values are represented as the means±SEM (n=10); Quantitative analysis of DCF in HK2 cells. Values are represented as the means±SEM of three replicates from three different experiments. SNx, 5/6 nephrectomy; PFD, pirfenidone. ^*^ *P*<0.05, SNx rats vs. sham or rotenone/CCCP/ H_2_O_2_ incubation vs. control; ^#^ *P*<0.05, SNx+PFD vs. SNx rats or PFD+rotenone incubation vs. rotenone incubation alone.

Electron microscopy of rats underwent 5/6 nephrectomy (SNx) revealed renal proximal tubular cells that contained numerous dysmorphic mitochondria, including swelling and disrupted cristae architectures. Some mitochondria exhibited disrupted membranes and the complete loss of cristae compared to normal mitochondria in the sham group. Few swollen mitochondria were observed after pirfenidone treatment, and most mitochondria exhibited less-damaged cristae structures (Figure 2 A). Pirfenidone protection against injury to tubular cell mitochondria was also investigated in vitro. HK2 cells were cultured in vitro. Mitochondria are the power stations of cells, and the respiratory chain is the preferential target of many xenobiotics. We used rotenone to damage HK2 cells and induce apoptosis, and similar mitochondrial changes were observed after incubation with the mitochondrial respiratory chain complex inhibitor (Figure 2 B). The mitochondrial injuries were assessed according to Flameng grading. The socres of mitochondria in renal proximal tubular cells were obviously lower than that of the control group in vivo and in vitro study ,and the scores were improved significantly after pirfenidone treatment (Figure 3 C). Pirfenidone relieved swollen mitochondria and protected the cristae architecture, which suggests its protection of mitochondrial structure. 

### Pirfenidone stabilized mitochondrial functions in renal proximal tubular cells

 A decline in mitochondria membrane potential (MMP) suggests damage to the mitochondrial membrane, which is indicated as a decrease in the red-to-green fluorescence ratio determined using JC-1. MMP in kidney cortexes deceased drastically compared to the sham group, and pirfenidone treatment significantly reduced MMP loss (Figure 3 A). A significant increase in MMP was observed after pirfenidone treatment compared to the disruption of MMP in the presence of rotenone for 48 h (Figure 3 B). 

ROS production was monitored using DCF fluorescence intensity. Isolated mitochondria from kidney cortexes in SNx rats exhibited a dramatic increase in ROS production, and pirfenidone treatment significantly reduced ROS production (Figure 3 C). Mitochondrial respiration is the major source of ROS. Therefore, rotenone was used to inhibit the mitochondrial respiratory chain. ROS production was remarkably enhanced after respiratory chain complex inhibition. Pirfenidone intervention significantly reduced the enhanced levels of intracellular ROS (Figure 3 C). The amount of H_2_O_2_ generated by HK2 cells was similar to the ROS detected above, which confirmed pirfenidone’s ability to decrease oxidants (Figure 3 D).

**Figure 4:**
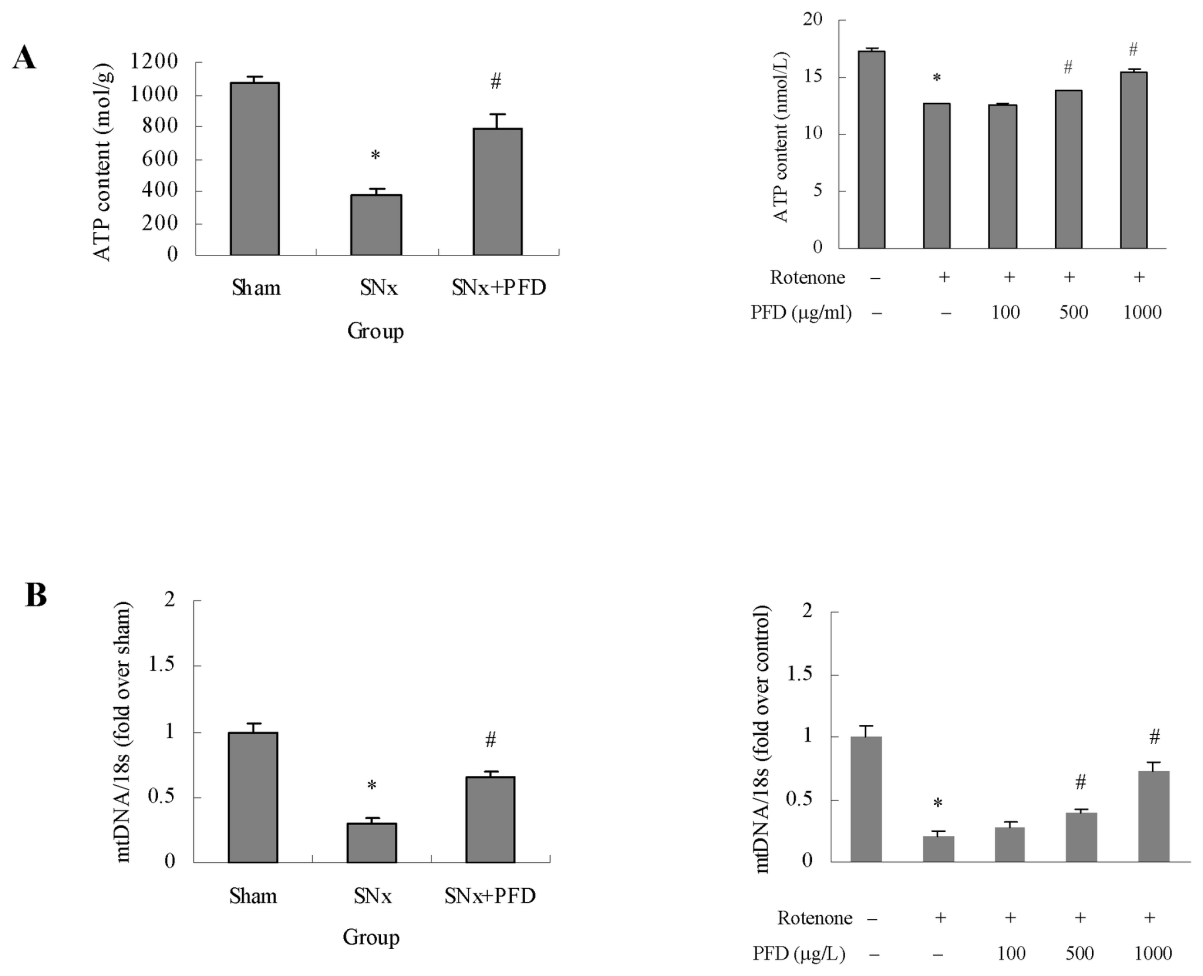
Pirfenidone increased the ATP content and stabilized the mitochondrial DNA (mtDNA) copy number in renal proximal tubular cells. (A) Quantitative analysis of ATP in kidney cortexes of rats. Values are represented as the means±SEM (n=10); Quantitative analysis of ATP in HK2 cells. Values are represented as the means±SEM of three replicates from three different experiments. (B) quantitative analysis of mtDNA in kidney cortexes of rats. Values are represented as the means±SEM (n=10); Quantitative analysis of mtDNA in HK2 cells. Values are represented as the means±SEM of three replicates from three different experiments. SNx, 5/6 nephrectomy; PFD, pirfenidone. rRNA, ribosomal RNA. ^*^ *P*<0.05, SNx rats vs. sham or rotenone incubation vs. control; ^#^ *P*<0.05, SNx+PFD vs. SNx rats or PFD+rotenone incubation vs. rotenone incubation alone.

ATP levels in remnant kidney cortexes were dramatically lower than sham levels, and these levels improved after pirfenidone treatment (Figure 4 A). Blockade of the mitochondrial respiratory chain also decreased ATP production in vitro, and pirfenidone treatment significantly improved ATP levels in a dose-dependent manner compared to the mitochondria injury groups (Figure 4 A).

 The mtDNA copy numbers in kidney cortexes were significantly decreased in SNx rats, and these numbers improved with pirfenidone treatment (Figure 4 B). We measured mtDNA in HK2 cells by damaging the mitochondrial respiratory chain to verify the sensitivity of the mtDNA copy number to pirfenidone. Rotenone severely decreased the mtDNA copy number, and pirfenidone intervention significantly increased the mtDNA copy number, which suggested pirfenidone stabilized mitochondrial functions in tubular cells (Figure 4 B).

### Pirfenidone suppressed renal interstitial fibrosis and proximal tubular cell apoptosis

**Figure 5:**
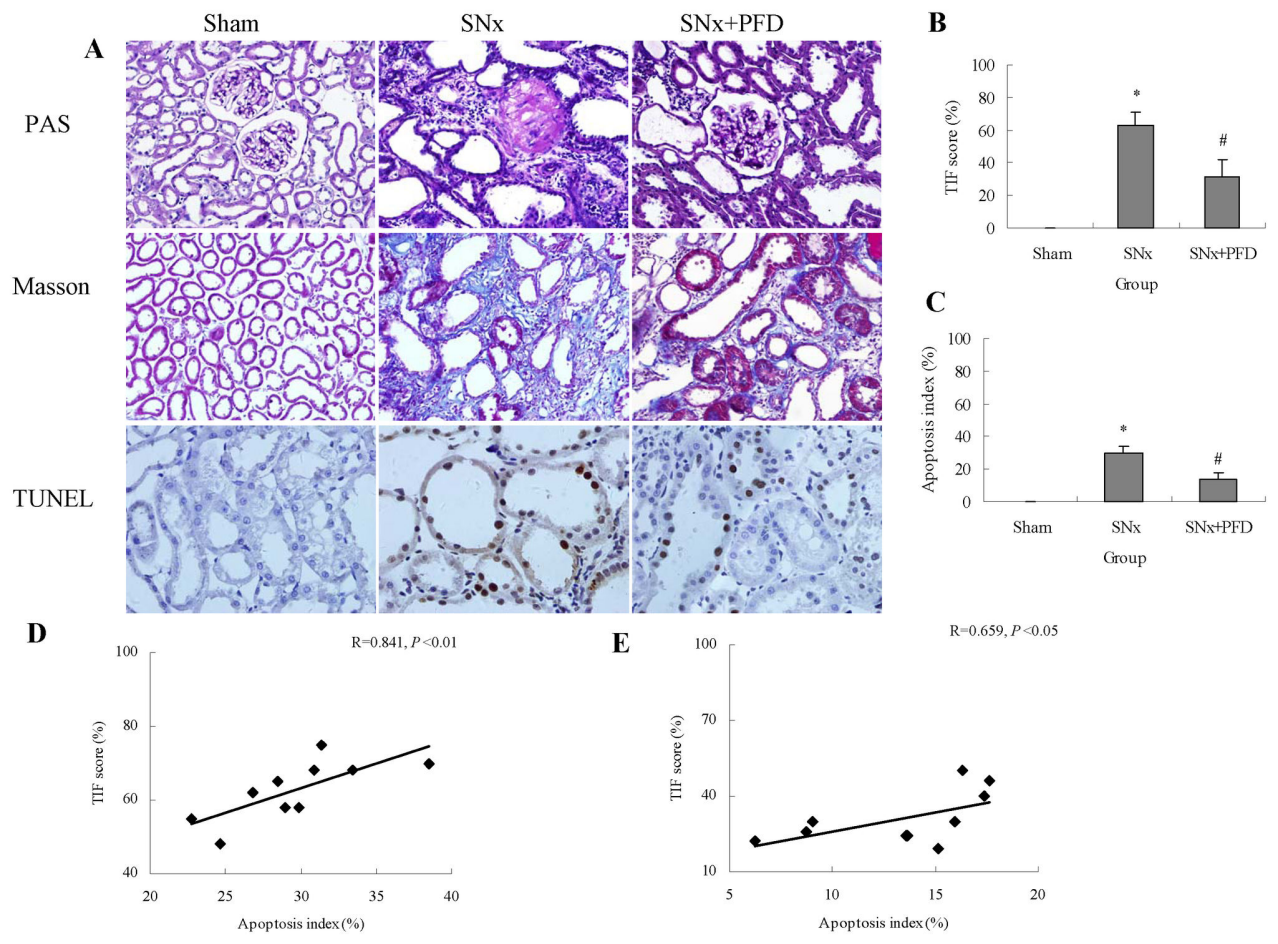
Pirfenidone ameliorated renal injuries including renal tubulointerstitial fibrosis and proximal tubular cell apoptosis in SNx rats. Representative light micrographs of renal tissues obtained from sham-operated, SNx and PFD-treated rats 12 weeks after renal ablation. A (200× magnification) is representative of periodic acid-schiff and masson trichrome staining (200× magnification) and TdT-mediated dUTP nick end labeling (TUNEL) (400× magnification). Renal tubulointerstitial fibrosis score (B) and proximal tubular cell apoptosis indexes (C) were calculated. Correlations between tubulointerstitial fibrosis and the apoptosis index were analyzed in SNx (D) and PFD-treated rats (E). SNx, 5/6 nephrectomy; PFD, pirfenidone; TIF, tubulointerstitial fibrosis. PAS, periodic acid-schiff. Values are represented as the means±SEM (n=10). * P<0.05, SNx vs. sham; # P<0.05, SNx+PFD vs. SNx rats.

 Kidney pathological changes were observed using periodic acid-schiff (PAS) and trichrome masson staining (Figure 5 A), which revealed that pirfenidone reduced kidney injuries including fibrotic lesions in SNx rats (Figure 5 B). We investigated the effect of pirfenidone treatment on renal proximal tubular cell apoptosis in vivo. The TUNEL assay demonstrated that proximal tubular cell apoptosis was reduced significantly in the pirfenidone-treated group (Figure 5 A and C). We conducted a correlation analysis between proximal tubular cell apoptosis and renal interstitial fibrosis because tubular cell apoptosis plays a critical role in the promotion of renal fibrosis progression. Our results revealed a positive correlation between the number of apoptotic cells and fibrosis with or without pirfenidone treatment (Figure 5 D and E). 

**Figure 6:**
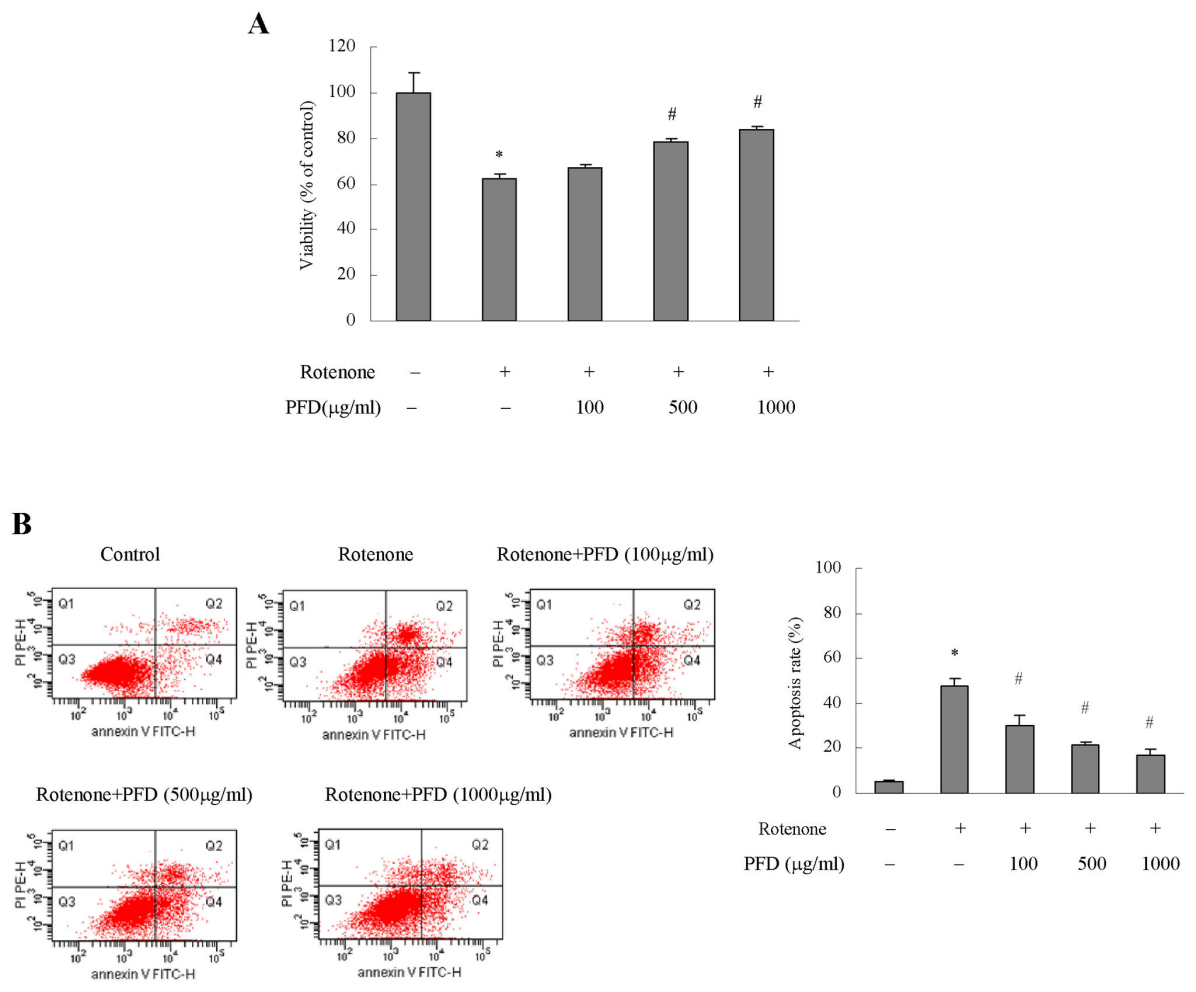
Pirfenidone sustained HK2 cells viability and inhibited their apoptosis after mitochondrial injury. Rotenone induced cell injury, and pirfenidone was used at 100, 500 and 1000 μg/ml (A). HK2 cell apoptosis was determined using annexin ν/PI staining and flow cytometry analysis (B). PFD, pirfenidone. Values are represented as the means±SEM of three replicates from three different experiments. ^*^ *P*<0.05, rotenone incubation vs. control; ^#^ *P*<0.05, PFD+rotenone incubation vs. rotenone incubation alone.

HK2 cells were cultured in vitro to further investigate the anti-apoptotic effects of pirfenidone in renal proximal tubular cells. HK2 cells viability deceased markedly after incubation with rotenone for 48 h, and pirfenidone intervention significantly prevented the decline in cellular viability in a dose-dependent manner (Figure 6 A). Cell apoptotic rates dramatically increased after using rotenone. Notably, pirfenidone significantly inhibited cell apoptosis in a dose-dependent manner (Figure 6 B). 

### Pirfenidone inhibited the mitochondria-mediated apoptosis signaling pathway in renal proximal tubular cells

**Figure 7:**
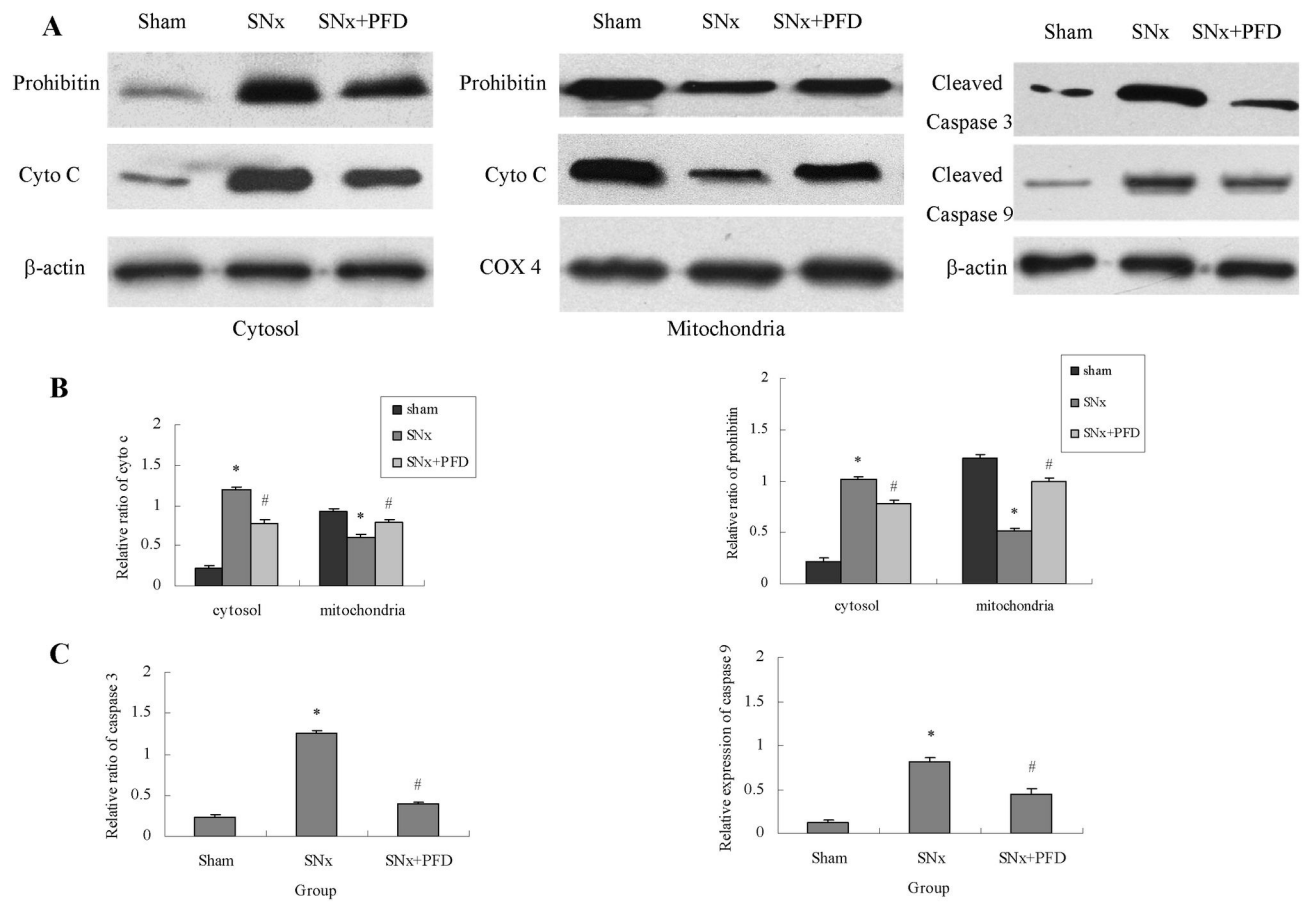
Effect of pirfenidone on cytochrome C, prohibitin and cleaved caspase-3/9 in kidney cortexes of rats. (A) Western blotting analysis for cyto C, prohibitin, cleaved caspase-3 and cleaved caspase-9. (B) Quantification of cyto C and prohibitin. (C) Quantification of cleaved caspase-3 and cleaved caspase-9. SNx, 5/6 nephrectomy; PFD, pirfenidone. Values were represented as the means±SEM, n=6. ^*^ *P*<0.05, SNx rats vs. sham; ^#^ *P*<0.05, SNx+PFD vs. SNx rats.

**Figure 8:**
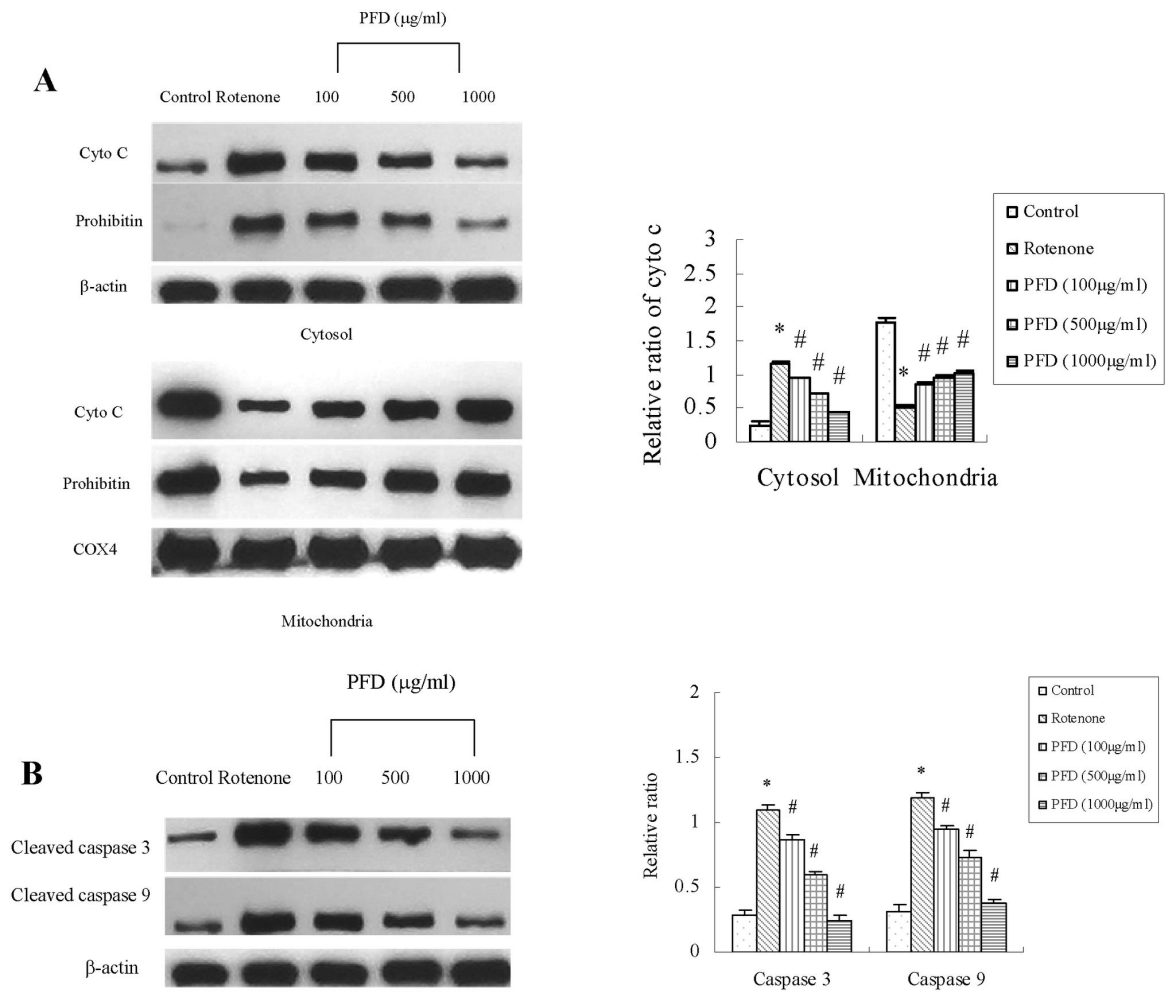
Effect of pirfenidone on cytochrome C, prohibitin and cleaved caspase-3/9 in HK2 cells. (A) Western blotting analysis for cyto C and prohibitin; Quantification of cyto C and prohibitin. (B) Western blotting analysis for cleaved caspase 3 and cleaved caspase 9; Quantification of cleaved caspase 3/9. PFD, pirfenidone; Values are represented as the means±SEM of three replicates from three different experiments. ^*^ *P*<0.05, rotenone incubation vs. control; ^#^ *P*<0.05, PFD+rotenone incubation vs. rotenone incubation alone.

The transcription of caspase-3 or caspase-9 was not altered by SNx (remnant kidney cortexes) or mitochondrial respiratory inhibition (HK2 cells) when tested in the absence or presence of pirfenidone. Cytosolic and mitochondrial fractions were isolated from remnant kidney cortexes and HK2 cells. Cytochrome C and prohibitin increased significantly in the cytosolic fractions from SNx rats compared to the sham group, which suggested mitochondrial leakage in SNx rats (Figure 7 A and B). Pirfenidone treatment significantly decreased prohibitin and cytochrome C content in the cytosolic fractions. Pirfenidone also prevented mitochondrial leakage in vitro compared to control groups treated with rotenone (Figure 8 A). 

 Caspase-3 and caspase-9 play important roles in the mitochondria-mediated apoptosis signaling pathway. The cleaving of caspase-3 and caspase-9 is critical for the activation of the signaling pathway. Pirfenidone significantly decreased cleaved caspase-3 and cleaved caspase-9 proteins compared to SNx rats (Figure 7 A and C). In HK2 cells, rotenone promoted the cleavage of caspase-3 and caspase-9, which suggested the activation of the apoptosis signaling pathway. Pirfenidone intervention significantly reduced cleaved caspase-3 and cleaved caspase-9 levels in vitro, which suggested that this drug inhibited the apoptosis signaling pathway (Figure 8 B). 

### Pirfenidone reduced oxidative stress in renal proximal tubular cells

**Figure 9:**
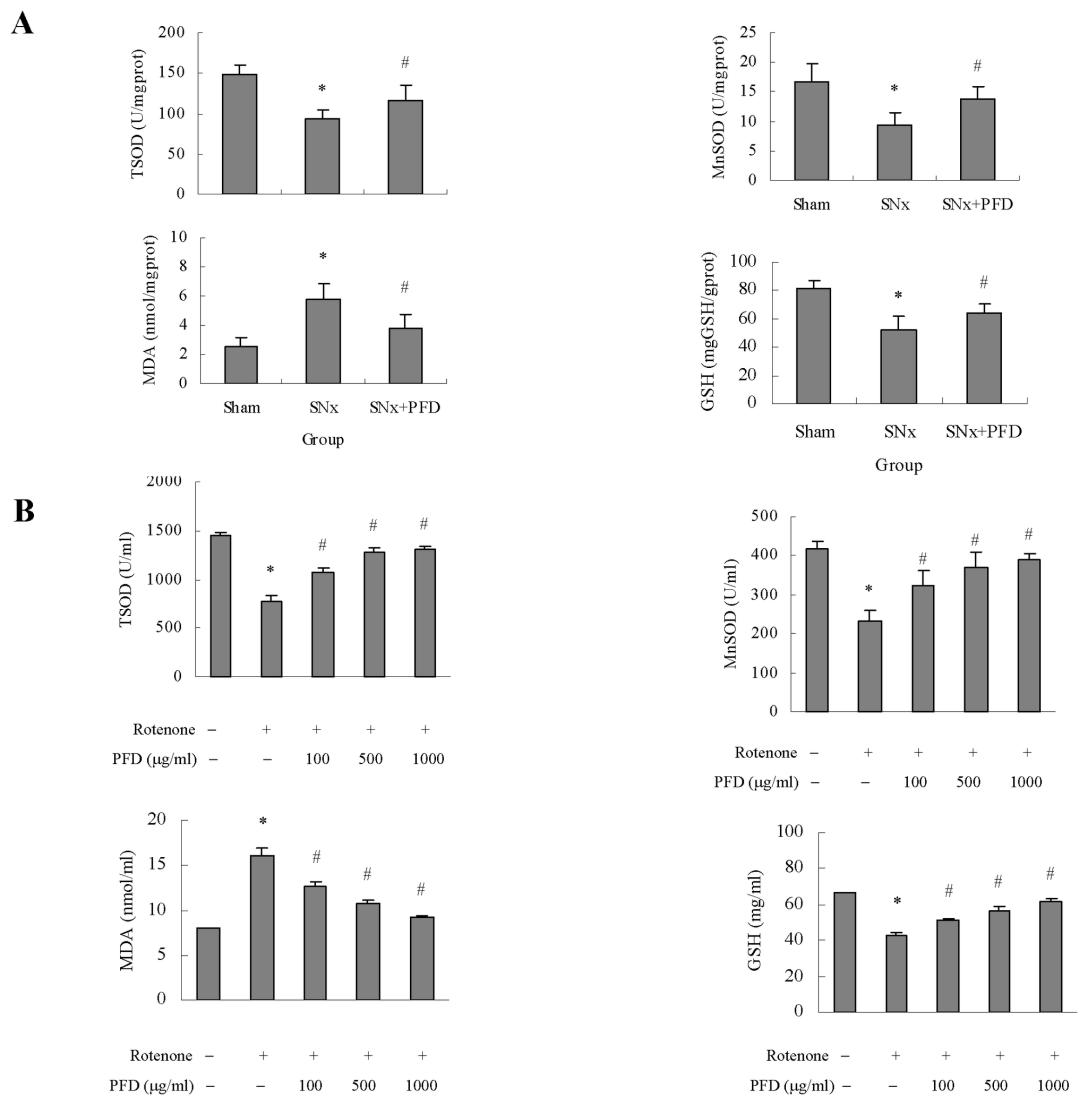
Evaluation of totoal SOD (TSOD), Mn-SOD, MDA and GSH activity by pirfenidone intervention. (A) Activities of TSOD, Mn-SOD, MDA and GSH in kidney cortexes of rats were displayed. Values were represented as the means±SEM, n=10; (B) Quantification of TSOD, Mn-SOD, MDA and GSH in HK2 cells. Values are represented as the means±SEM of three replicates from three different experiments. SNx, 5/6 nephrectomy; PFD, pirfenidone; SOD, superoxide dismutase. Mn-SOD, Manganese superoxide dismutase. MDA, malondialdehyde; GSH, glutathione. ^*^ *P*<0.05, SNx rats vs. sham or rotenone incubation vs. control; ^#^ *P*<0.05, SNx+PFD vs. SNx rats or PFD+rotenone incubation vs. rotenone incubation alone.

 Major antioxidant defenses include antioxidant scavengers, such as total SOD and GSH. An increase in MDA, which is a lipid peroxidation end-product, indicates reduced antioxidant capacity. We measured total SOD, GSH and MDA activities in remnant kidney cortexes and HK2 cells. The total SOD activity was decreased in SNx rats, and the total SOD activity was reduced after mitochondrial respiratory complex inhibition in HK2 cells. Mn-SOD exists in mitochondria, and it indicates the antioxidant activity of mitochondria. We also calculated the Mn-SOD levels based on total SOD and demonstrated that Mn-SOD was also decreased in vivo and in vitro, which was consistent with the behavior of the total SOD activity. Pirfenidone significantly increased SOD and Mn-SOD (Figure 9 A and B). The MDA activity was notably enhanced in SNx rats, and the inhibition of the mitochondrial respiratory chain in vitro promoted MDA activity. Pirfenidone treatment significantly decreased MDA activity in vivo and in vitro (Figure 9 A and B). Pirfenidone also significantly enhanced GSH content in vivo and in vitro (Figure 9 A and B).

## Discussion

This study demonstrated that pirfenidone exerted its anti-tubulointerstitial fibrosis activity via the attenuation of mitochondrial dysfunction in renal proximal tubular cells and thus, inhibited apoptosis and oxidative stress.

Pirfenidone has been used clinically for the treatment of various fibrotic diseases, including idiopathic pulmonary fibrosis [15], multiple sclerosis [16], liver fibrosis [17], and fibrotic renal diseases [18]. Takakuta et al found that it inhibited TGF-β induced expression of fibronectin in NRK52E cells [19], and it has been verified to reduce in vitro rat renal fibroblast activation and mitogenesis [20]. However, the precise anti-fibrosis mechanism of this drug is not clear. Our previous study in the same animal model found that fibronectin was decreased significantly after pirfenidone treatment [21]. The present study successfully demonstrated that pirfenidone improved renal functions and ameliorated tubulointerstitial fibrosis by preventing mitochondrial dysfunction in renal tubular cells.

Persistent tubular injuries lead to Extracellular matrix (ECM) accumulation in tubulointerstitium, and facilitate the progression of renal fibrosis [22,23]. The proximal tubules rely heavily on aerobic metabolism for the production of ATP by oxidative phosphorylation because these structures exhibit minimal glycolytic capacity [24]. Mitochondrial damage generally occurs soon after injury in the proximal tubules and ATP depletion leads to cell loss, which reduces renal function and promotes renal fibrosis. Corcoran [25] et al demonstrated that mitochondria amplify TGF-β1-induced fibrotic responses in renal epithelial cells, suggesting mitochondria contribute to the progression of renal fibrosis by modulating TGF-β1 signal transduction. Pirfenidone treatment preserved the cristae structure in most of the mitochondria in proximal tubular cells, and fewer swollen mitochondria were observed in pirfenidone-treated rats compared to SNx rats. These results suggested that pirfenidone protected the mitochondrial structure in the proximal tubules. The pirfenidone molecule is able to move through cell membranes without requiring a receptor [26]. Although the mechanism of how it targets the mitochondria is still unclear, its treatment markedly improved mitochondrial damage, which suggested its protective effects in the mitochondrial structure of proximal tubular cells. 

 Alterations in the mitochondrial structure are generally related to the mitochondrial function. The protective effect of pirfenidone on mitochondrial function was determined using the mitochondrial membrane potential (MMP), mitochondrial DNA (mtDNA) copy number and ATP production. This study inhibited the mitochondrial respiratory chain in HK2 cells reduced MMP, which is an early characteristic feature of mitochondrial injury. Pirfenidone significantly prevented the decline in MMP. mtDNA damage produces defects in mtDNA-encoded gene expression and respiratory chain complex enzymes, which contributes to the loss of mitochondrial biogenesis. Pirfenidone treatment in vivo and in vitro significantly prevented the decline in the mtDNA copy number, which suggested the role of mtDNA in the maintenance of normal mitochondrial function. ATP recovery by pirfenidone may rescue tubular cells from reversible cellular swelling and prevent cell injury. Thus, pirfenidone prevented declines in mitochondrial functions in proximal tubular cells. 

 Mitochondrial dysfunction initiates the apoptotic signaling pathway, and mitochondria are the central executioners of apoptosis. Tubular cell apoptosis is an early event that occurs prior to the onset of renal fibrosis [27], and results in matrix synthesis and deposition in the renal interstitium. The translocation of cytochrome C from mitochondria to the cytosol is a crucial step in the activation of the apoptotic machinery in various cell death models [28]. Cytochrome C and caspase activation were accompanied with albumin-induced apoptosis [29]. In our study, pirfenidone treatment prevented translocation of cytochrome C and prohibitin from mitochondria into cytosol, resulting in inhibiting of caspase-9 and caspase-3 cleavage. Pirfenidone inhibited apoptosis in tubular cells by maintaining mitochondrial membrane stability, which subsequently inhibited the mitochondrial apoptotic signaling pathway. 

The clinical relevance of mitochondrial dysfunction and oxidative stress in progressive renal disease has been highlighted by recent studies in which antioxidant therapeutics that specifically target mitochondrial ROS ameliorate diabetic nephropathy and tubulointerstitial fibrosis lesions in experimental models [30]. Mitochondria constitute the primary cellular source of ROS [31], and the use of pirfenidone in vivo and in vitro decreased ROS production and hydrogen peroxide release. Suppression of ROS formation attenuated renal interstitial fibrosis and tubular apoptosis in db/db transgenic mice [32], and catalase deficiency increased mitochondrial ROS accompanied with renal fibrosis in diabetic nephropathy mice [33]. These results suggested that pirfenidone exerted anti-oxidative properties through mitochondrial mediation. Pirfenidone prevented the decline in Mn-SOD, which is an anti-oxidant in mitochondria that counteracts the generation of ROS. This result supported the anti-oxidant effects of pirfenidone as a mechanism of mitochondrial protection. Therefore, the anti-oxidant properties of pirfenidone may be partially via protection of mitochondria in tubular cells. 

**Figure 10:**
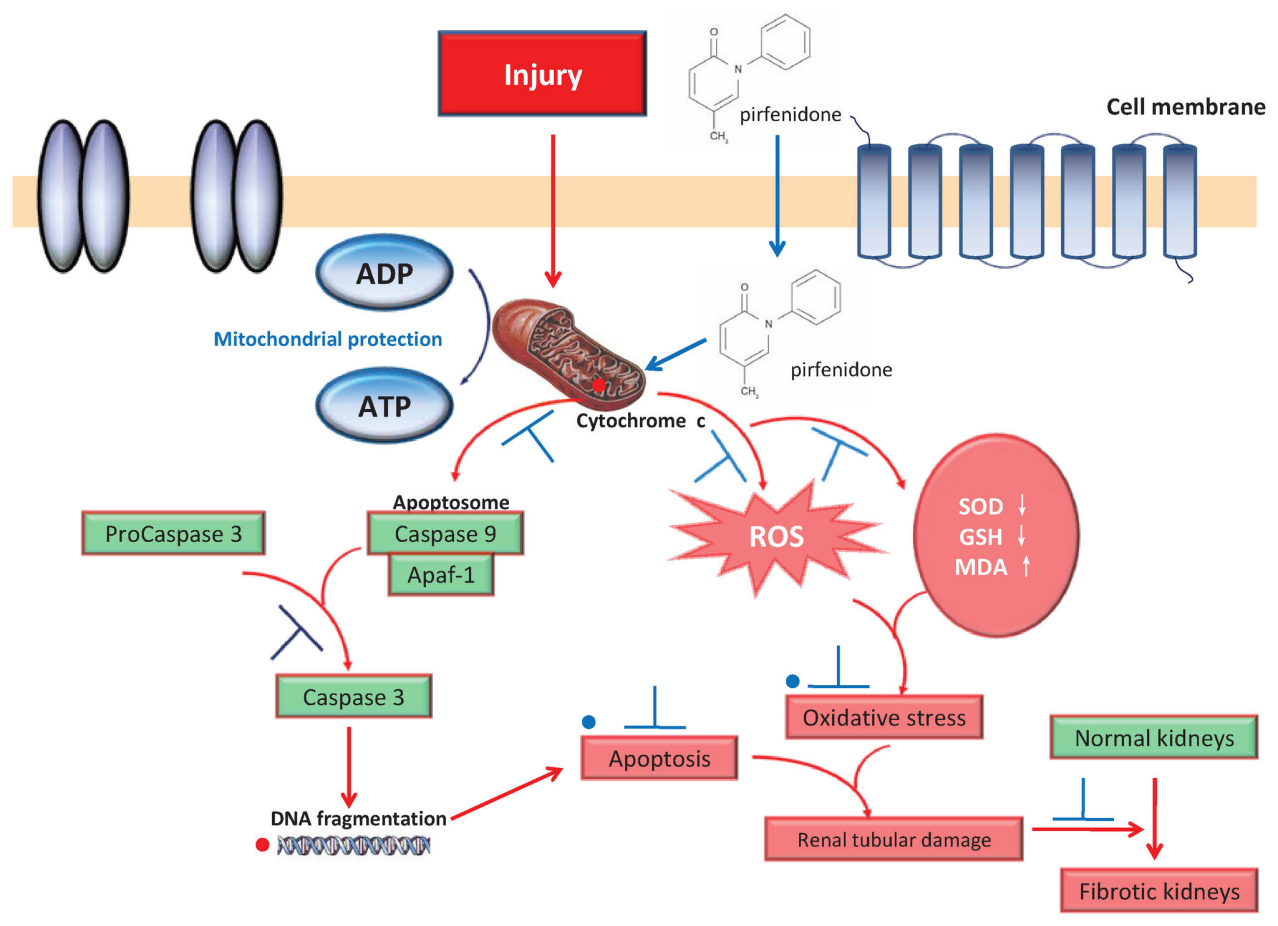
Pirfenidone’s protection of mitochondria in renal proximal tubular cells.

 In summary, our results demonstrated that pirfenidone protected the mitochondria of renal proximal tubular cells. This drug decreased apoptosis and ameliorated oxidative stress in proximal tubular cells at least partially via the stabilization of mitochondrial structures and functions, which improved renal function and inhibited renal fibrosis (Figure 10). Pirfenidone may be effective in the earlier stages of CKD prior to onset of renal fibrosis, because mitochondrial damage occurs quickly after kidney injury. These results provide new insights into the anti-fibrotic mechanisms of pirfenidone.
